# Mixed Trophoblastic Tumour After Early Pregnancy Loss and Spontaneous Conception During Surveillance: A Case Report

**DOI:** 10.7759/cureus.94608

**Published:** 2025-10-15

**Authors:** Shahela Nasir, Fiona Harris, Fusun Sirkeci, Uzma Kashif

**Affiliations:** 1 Obstetrics and Gynaecology, Wythenshawe Hospital, Manchester, GBR

**Keywords:** choriocarcinoma, epithelioid trophoblastic tumour, gestational trophoblastic neoplasia, mixed gestational trophoblastic tumour, placental site trophoblastic tumour

## Abstract

Mixed trophoblastic tumours (MTTs), which include choriocarcinoma and intermediate trophoblastic tumours like placental site trophoblastic tumour (PSTT), are very uncommon types of gestational trophoblastic neoplasia (GTN). Diagnosing and treating them continues to be challenging, especially when preserving fertility is important. A 37-year-old woman (G2P1) reported with a positive pregnancy test, abdominal pain, and brown vaginal discharge at 3+6 weeks of gestation. Initial examinations revealed variable serum β-hCG levels and ultrasound evidence of early intrauterine pregnancy. Followed by spontaneous miscarriage, a histology analysis of the products of conception showed an MTT (choriocarcinoma and PSTT). Staging CT and MRI showed no metastases, and β-hCG levels returned to normal. The patient was recommended for a hysterectomy with ovarian preservation. A second opinion confirmed this, but she wants to preserve her fertility. Later, a hysteroscopic-guided biopsy showed no signs of residual disease, and a follow-up was set up. During follow-up, she became pregnant spontaneously and is now continuing the care under a consultant-led maternal medicine team. This case highlights the diagnostic complexity of MTTs, the interplay between oncological safety and fertility preservation, and the necessity of multidisciplinary consultation.

## Introduction

We present a case regarding a mixed gestational trophoblastic tumour (choriocarcinoma and placental site trophoblastic tumour (PSTT)) subsequent to a miscarriage. Preliminary examinations indicated normal serum human chorionic gonadotropin (hCG) levels and an absence of metastases. Despite clinical recommendations for a hysterectomy, the patient declined definitive management due to her desire for fertility and subsequently conceived spontaneously. This case illustrates the diagnostic challenges, the intricate relationship between oncological guidelines and fertility preservation, and the significance of collaborative decision-making in rare gestational trophoblastic neoplasia (GTN).

A rare category of pregnancy-related tumours known as gestational trophoblastic neoplasia is caused by abnormal trophoblastic tissue proliferation. Invasive mole, choriocarcinoma, PSTT, and epithelioid trophoblastic tumour (ETT) are among the entities that make up GTN. Each of these tumours possesses unique biological features and clinical manifestations. In contrast to PSTT and ETT, which grow slowly and frequently present with low or normal hCG and limited chemosensitivity, choriocarcinoma is typically aggressive, highly vascular, and linked to very high hCG levels. The diagnosis and course of treatment are significantly impacted by these variances.

Less than 1% of all GTN cases are mixed trophoblastic tumours (MTTs), which are extremely rare and involve two or more histologic subtypes in a single lesion. Their diverse nature makes diagnosis challenging because treatment must be tailored to the predominant component. The occurrence of spontaneous conception during post-diagnostic surveillance, which is rarely documented in the literature, contributes to this case being unusual. It draws attention to the biological uncertainty of mixed GTNs and the significance of multidisciplinary, patient-centred care in guiding therapeutic choices.

## Case presentation

A 37-year-old woman with a history of one vaginal delivery presented to the gynaecological emergency unit. She had a positive urine pregnancy test and a brief history of right-sided rib pain radiating to the back, mild shoulder pain, intermittent light-headedness, and brown vaginal discharge. She was 3+6 weeks from her last menstrual period. She had a history of autoimmune skin disease that was treated with topical steroids and transient anxiety. The cervical smears were normal and up to date. She did not smoke or drink, and she lived with her partner and child.

**Figure 1:**
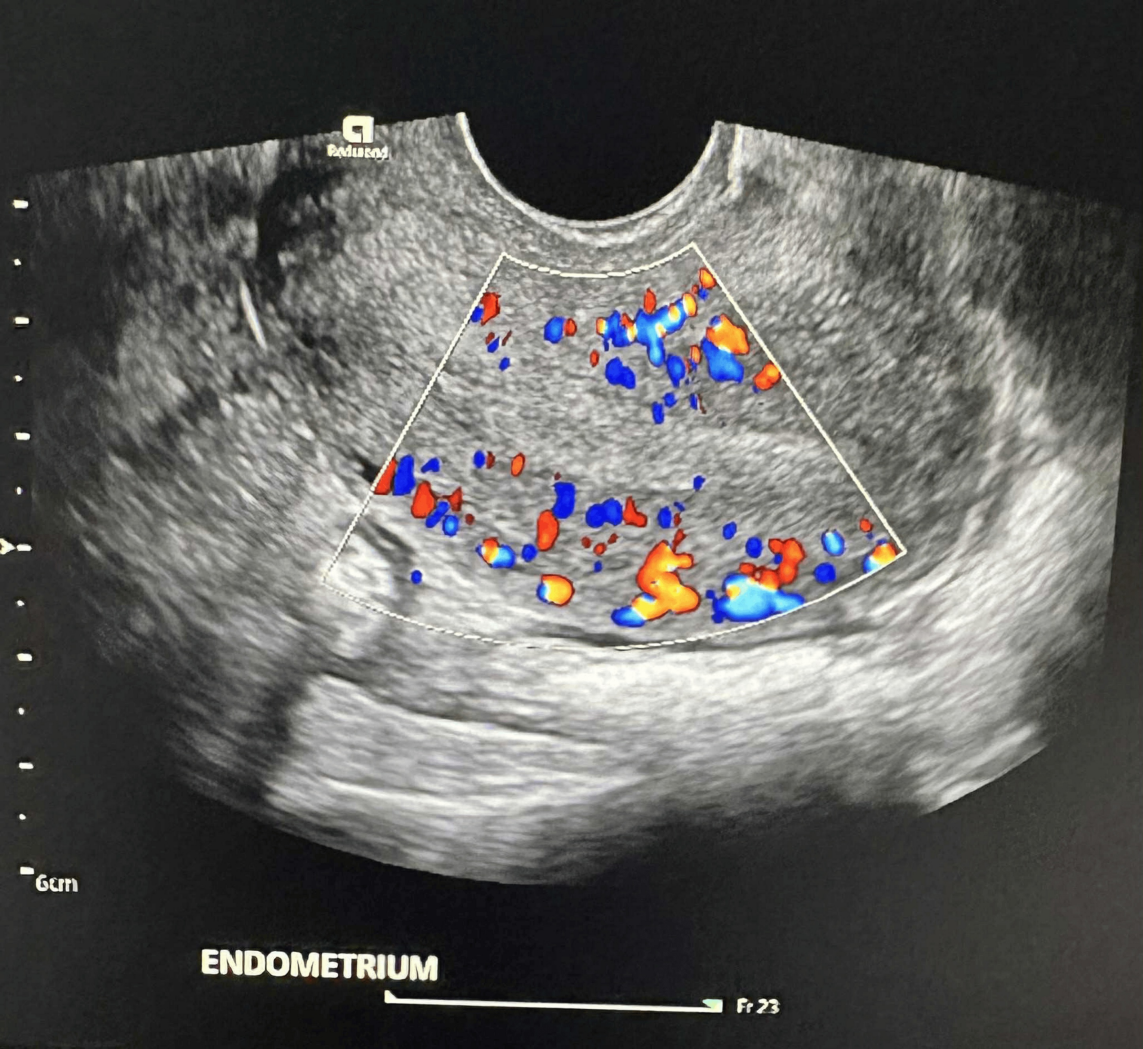
US Doppler post-miscarriage Subsequent imaging after passage of pregnancy tissue showed an empty uterus with normal endometrial lining.

Initial investigations indicated an increase in serum β-hCG from 497 IU/L to 3635 IU/L, followed by a decline to <1 IU/L, with the result remaining negative thereafter (Table 1). Ultrasound showed a single intrauterine gestational sac. No adnexal pathology or free fluid was detected. Subsequent imaging after passage of pregnancy tissue showed an empty uterus with normal endometrial lining (Figure 1). Histological analysis of the products of conception, obtained post-spontaneous miscarriage, confirmed an MTT, specifically choriocarcinoma and PSTT. She was sent to the national trophoblastic disease centre for further treatment. Staging tests, including a chest and abdominal CT scan and an MRI of the brain and pelvis, showed no signs of metastases or ongoing disease.

**Table 1:** Laboratory investigations were within normal limits; β-hCG remains negative after miscarriage β-hCG: beta-human chorionic gonadotropin; Plts: platelets

Parameters	Result	Reference Range	Unit
WBC	6.9	4.0–11.0	10⁹/L
Haemoglobin	126.0	115.0–165.0	g/L
Plts	244.0	150.0–400.0	×10⁹/L
Creatinine	61	45–84	µmol/L
ALT	13	0–21	µmol/L
β-hCG	497 --> 1199 -->3635--> <1	0–4	IU/L

She received extensive counselling about the diagnosis and was informed that the international standard of care for stage I MTTs is hysterectomy with ovarian preservation. The prognostic importance of the duration since her last completed pregnancy, which exceeded a decade, was emphasised, indicating a possible necessity for adjuvant chemotherapy. An antecedent pregnancy lasting more than four years is recognised as an adverse prognostic risk factor. Post-hysterectomy follow-up imaging suggested an MRI every six months for the first year and then once a year for the next five years, as well as chest imaging.

The patient was devastated about the diagnosis and the recommendation of definitive surgery due to the risk of residual microscopic disease. The patient explored fertility-sparing options and experimental treatments, such as pembrolizumab immunotherapy, but these were not recommended. A second opinion from a European Multidisciplinary Team (MDT) meeting made it clear that the standard care recommendation would be hysterectomy, which is internationally agreed upon. Her hCG was <1 IU/L; the CT chest and abdomen showed no evidence of metastases; the MRI brain showed no evidence of intracranial mass; and the MRI pelvis did not show any obvious evidence of disease according to MRI criteria.

A subsequent hysteroscopic endometrial biopsy was performed, and the histology showed multiple fragments of inactive endometrial tissue. A surveillance MRI of the pelvis was requested for monitoring. During follow-up, the patient conceived spontaneously and is presently continuing with an intrauterine pregnancy, with an estimated delivery date in January 2026. Her pregnancy was regarded as a high-risk pregnancy and a possible malignancy. Her care has been dedicated to consultant-led maternal medicine, with recommendations for thromboprophylaxis. The patient would like a completion hysterectomy following delivery of the baby.

## Discussion

GTN refers to a collection of uncommon malignant tumours originating from trophoblastic proliferation affecting approximately 20,000 women per year worldwide. This includes invasive mole, choriocarcinoma, and intermediate trophoblastic tumours (ITTs), such as PSTT and ETT [1]. Although these entities originate from trophoblastic tissue, their diagnosis and management exhibit considerable differences. Choriocarcinoma is typically diagnosed based on clinical presentation and significantly elevated serum hCG levels; thus, histological confirmation is not routinely necessary [2]. The condition exhibits significant chemosensitivity, with first-line chemotherapy resulting in a cure for the majority of cases [3].

Mixed GTN is a rare condition defined by the simultaneous presence of various trophoblastic tumours [3]. The diagnosis and management of mixed GTN continue to be difficult because it is rare and biologically heterogeneous, underscoring the necessity of early identification and referral to specialised centres for optimal management [1]. Table 2 presents the features of the different GTN subtypes.

**Table 2:** Features of gestational trophoblastic neoplasia (GTN) subtypes

Feature	Choriocarcinoma (CC)	Placental Site Trophoblastic Tumour (PSTT)	Epithelioid Trophoblastic Tumour (ETT)	Mixed Trophoblastic Tumour (MTT)
Incidence	Rare (~5% of GTN)	Very rare (1–2% of GTN)	Extremely rare (<1% of GTN)	Extremely rare (case reports only)
Origin	Cytotrophoblast & syncytiotrophoblast	Implantation-site intermediate trophoblast	Chorionic-type intermediate trophoblast	Combination of CC + PSTT/ETT
hCG levels	Markedly elevated	Normal or mildly elevated	Normal or mildly elevated	Variable
Spread/growth	highly metastatic (lung, brain, liver common) Rapid	Locally invasive (uterus, nodes), slow	locally invasive (uterus, nodes) Slow	Behaviour depends on the dominant element
Chemosensitivity	Very high (EMA/CO or single-agent if low risk)	Poor (limited response)	Poor (limited response)	Mixed
Standard treatment	Chemotherapy	Hysterectomy (± chemo)	Hysterectomy (± chemo)	Surgery ± chemo depending on histology
Prognosis	Excellent with chemo (>90% cure)	Good if localised and resected	Good if localised and resected	Variable; worse than pure forms, depends on the stage

PSTT and ETT can occur after any previous pregnancy and usually show normal or only slightly elevated levels of serum hCG because there is no syncytiotrophoblast [4]. These tumours exhibit relative resistance to chemotherapy, and hysterectomy remains the primary treatment for cases localised to the uterus, with chemotherapy reserved for metastatic or high-risk disease [5].

This case report indicates that PSTT, compared with choriocarcinoma, is a slower-growing tumour and that these tumours often do not secrete hCG, making it an unreliable biomarker. Slow-growing tumours can be more difficult to treat in terms of chemotherapy responses and may develop mutations over time. This is probably why the interval between the antecedent pregnancy and development of PSTT, particularly when greater than four years, is a significant prognostic factor [6].

Overall, GTN is highly chemosensitive, with cure rates of approximately 80-90%, even in high-risk scenarios [6]. Nevertheless, in individuals with chemoresistant or relapsed disease, outcomes remain poor, necessitating the development of innovative therapeutic approaches. The introduction of immunotherapy for solid tumours in gynaecological oncology has now been extended to GTN, with growing evidence supporting its application in high-risk and refractory settings [7].

Pembrolizumab is the most widely studied immunotherapy in this context. It functions by inhibiting programmed cell death protein-1 (PD-1) receptors on lymphocytes [8]. Emerging evidence indicates that pembrolizumab is effective in multi-drug-resistant GTN and appears to show activity in PSTT and ETT, particularly in relapse [9,10]. These promising results suggest that pembrolizumab may provide sustained remission and possibly a cure for patients with aggressive GTN resistant to conventional chemotherapy.

Due to their various presentations, mixed and ITTs are uncommon and challenging to diagnose. It highlighted the importance of molecular genotyping in confirming gestational origin [11]. This strategy was supported by the FIGO 2021 guidelines [12], which suggested surgery for PSTT and ETT and risk-adapted chemotherapy for choriocarcinoma.

Emerging treatments, such as immune checkpoint inhibition, hold promise for treating resistant GTN and enhancing conventional therapies [13]. Case-based reports emphasised the significance of histology and surgical excision, as well as the characteristic low hCG levels of PSTT [14], while other reports demonstrated that the approach should be customised to the dominant component, with mixed tumours frequently requiring surgery after chemotherapy failure [15,16]. While noting the risk of recurrence, when taken as a whole, these studies show that the key to improving outcomes for this uncommon class of tumours is precise diagnosis, tailored treatment, and interdisciplinary care (Table 3) [17].

**Table 3:** Literature review of mixed and ITT-predominant gestational trophoblastic neoplasia GTN = Gestational trophoblastic neoplasia; MTT = Mixed trophoblastic tumour; CC = Choriocarcinoma; PSTT = Placental site trophoblastic tumour; ETT = Epithelioid trophoblastic tumour; EMA/CO = Etoposide, methotrexate, actinomycin-D/cyclophosphamide, vincristine (standard multi-agent chemotherapy regimen)

Author/Year	Publication	Tumour Type	Management	Findings/Conclusion
Niu et al., 2025 [11]	Case series (3 patients)	CC + PSTT/ETT	Hysterectomy, multi-agent chemotherapy, and immunotherapy in recurrence	Diagnosis is often missed initially; DNA genotyping confirmed gestational origin; outcome depends on the dominant component; immunotherapy is effective in resistant cases.
Ngan et al., 2021 [12]	Guideline (all ages)	GTN (mole, CC, PSTT, ETT)	Risk-adapted chemotherapy, hysterectomy for ITT, MDT care	FIGO 2021 update: standardised global recommendations for diagnosis and management of GTD/GTN.
Baas, et al. 2023 [13]	Review (immunotherapy)	GTN incl. PSTT/ETT	Pembrolizumab (PD-1 inhibitor)	Described as a new paradigm; durable responses in resistant GTN.
Zampacorta C, et al. 2023 [14]	Case report	PSTT	Hysterectomy	Highlighted low β-hCG, histology as a key diagnostic tool, and surgery as a mainstay, with a risk of recurrence in ~15%.
Kong Y, et al. 2019 [15]	Case series (16 patients)	Mixed GTN (CC ± PSTT/ETT)	Surgery ± chemotherapy	Management depends on the dominant component; MTT is a very rare presentation.
Tse KY, et al. 2018 [16]	Case report	2 pure ETT; 3 mixed ETT + CC	Fertility-sparing surgery or hysterectomy; chemo given in mixed cases	All patients survived; recurrence is possible even years later; fertility was preserved in one case; mixed tumours required hysterectomy after chemo failure.
Gari 2015 [17]	Case report (postpartum)	CC + PSTT	Surgery ± chemo	Emphasised tailoring treatment to the dominant tumour and stage.

## Conclusions

Formulating an ideal treatment strategy for MTTs is difficult to accomplish due to various factors. To begin with, these tumours are very rare. Consequently, comprehension of their behaviour and therapeutic responsiveness is restricted. Additionally, the elements of MTTs exhibit varying responses to therapy. In particular, choriocarcinoma is very responsive to chemotherapy, but ETTs and PSTTs are chemoresistant. Total hysterectomy remains the mainstay for primary treatment with an excellent prognosis. Pembrolizumab appears to be an efficacious immunotherapy for patients with high-risk GTN exhibiting chemoresistant or relapsed disease, including cases of PSTT and ETT. Further studies are necessary to determine the role of immunotherapy.

The relationship between diagnostic results and management choices was clarified in this instance by a chronological timeline that summarised significant clinical events, from the patient's first presentation to the current pregnancy. In particular, the decision to pursue surveillance instead of surgery was supported by β-hCG normalisation and negative imaging, which reflected the patient's well-informed choice to preserve fertility. We hope for a safe pregnancy and a healthy outcome for both the mother and the child. Follow-up care and MDT input will help us determine further management. However, the patient remains committed to pursuing definitive management in the form of a hysterectomy following delivery.
